# Healthy Volunteer Studies in the Development of Anticancer Drugs with Genotoxic Findings

**DOI:** 10.1007/s43441-021-00330-8

**Published:** 2021-08-11

**Authors:** Grace Omes-Smit, Marjolein Garsen, Alex Zwiers

**Affiliations:** Zwiers Regulatory Consultancy, Gasstraat 20, 5349 AA Oss, The Netherlands

**Keywords:** Genetic toxicology, Genotoxicity testing, Oncology, Drug development, Clinical trials, Healthy volunteers

## Abstract

**Background:**

Recent scientific advances in cancer research have led to the development of immunomodulatory and molecularly targeted drugs with better safety profiles than chemotherapeutics, which makes it possible to include healthy volunteers (HVs) in clinical trials. In this study, we aimed to identify the number of marketing authorization applications (MAAs) that enrolled HVs in a clinical trial and to identify the number of anticancer drugs that were given to HVs despite a positive genotoxic finding. In addition, we evaluated the dose of anticancer drugs administered to HVs and the justification for proceeding with HV studies despite a positive genotoxic finding.

**Methods:**

Publicly available information from the European Medicines Agency (EMA) website was used for this study. Anticancer drugs were identified using the human medicines highlights published by EMA between January 2010 and December 2019. EPARs were used to collect general information of the anticancer drugs, details on genotoxicity studies, and the enrollment of HVs in clinical trials.

**Results:**

We identified 71 MAAs for small molecule anticancer drugs with a positive or negative CHMP opinion in the EU. Forty-eight anticancer drugs were studied in HVs, of which 12 anticancer drugs were administered to HVs despite positive genotoxic findings in the standard battery. Systematic and extensive genetic toxicology screening demonstrated the absence of genotoxic risks to the cell system.

**Conclusion:**

We showed that despite a positive genotoxic finding, comprehensive genetic toxicology testing demonstrated the absence of risks to the cell system at the human exposure dose. Therefore, these anticancer drugs posed no harm to HVs.

## Introduction

Genetic toxicology is the study of substances that cause deoxyribonucleic acid (DNA) damage, the mechanisms of the DNA damage, and the response of the cell or animal system to such damage [1]. Various in vitro (e.g., Ames test, chromosome aberration test, mouse lymphoma assay) and in vivo* (*e.g., micronucleus assay, chromosome aberration test) tests can be used to examine whether a chemical substance has the potential to cause DNA damage, which is important, as DNA damage may eventually lead to the development of cancer and/or other genetic defects [2, 3]. Genotoxicity studies are started during the non-clinical phase of the development of drugs. Before the start of phase I single-dose clinical trials, a gene mutation assay should be performed; and before the start of phase I multiple dose clinical trials, an assay capable of detecting chromosomal damage in a mammalian system should be complete [4]. A complete battery of genotoxicity tests should be completed before the initiation of phase II clinical trials [4]. Genotoxicity studies are not always required before the start of phase I clinical trials. For example, genotoxicity studies are not considered essential to support clinical trials for therapeutics intended to treat patients with advanced cancer [5]. As a result, in some situations, compounds which have a genotoxic potential can still proceed to first in human (FIH) trials.

FIH and phase I clinical trials are most often performed in healthy volunteers (HVs) but can be performed in patients as well. Traditionally, FIH and phase I clinical trials of anticancer drugs have been performed in patients, as in the past anticancer drug development mainly focused on chemotherapeutics, for which it is unethical to give to HVs because of their cytotoxic potential [6]. Recent scientific advances in cancer research have led to the development of immunomodulatory and molecularly targeted drugs with significantly lower toxicity and better safety profiles, which makes it possible to include HVs in clinical trials of anticancer drugs [6]. There are several practical advantages of including HVs instead of patients in clinical trials of anticancer drugs. These include lower costs, rapid enrollment, lower dropout rates, a homogenous study population (i.e., minimal confounding by comorbidities and/or concomitant medications), and better participant compliance resulting in fewer protocol deviations [6]. Disadvantages of the inclusion of HVs are that the pharmacokinetic (PK) properties of the drug may differ between healthy volunteers and patients, pharmacodynamic (PD) measurements may be of limited use and the target related to safety may be different in patients [6].

Several studies have reported an increase in the enrollment of HVs in phase I clinical trials of anticancer drugs [6–9]. To decide whether it is appropriate to conduct a FIH or early phase I clinical trial in HVs, developers take the potential for genotoxicity and the predicted starting dose of the anticancer drug into account [7]. A strong case can be made to conduct FIH or early phase I clinical trials in HVs if the preclinical efficacious dose is equal to or less than the no observed adverse effect level (NOAEL) and the drug is non-genotoxic [7]. The aim of this study was to identify the number of marketing authorization applications (MAAs) that enrolled HVs in a clinical trial and to identify the number of anticancer drugs that were given to HVs despite a positive genotoxic result in the European Union (EU) between January 2010 and December 2019. In addition, we evaluated the dose of the anticancer drugs with a positive genotoxic result administered to HVs and the justification for proceeding with HV studies despite a positive genotoxic result.

## Methods

Publicly available information from the website of the European Medicines Agency (EMA) was used for this study. Details on the identification of the anticancer products have been described previously [10]. In brief, anticancer drugs were identified using the human medicines highlights published by EMA in the period of January 2010 up to and including December 2019 [11]. Products under the heading cancer with a positive or negative CHMP opinion were analyzed and included in this study when they met the following criteria: (i) article 8(3) full or full-mixed application as legal basis; (ii) small molecule; (iii) new active substance or known active substance; (iv) products developed for the treatment of cancer (excluding products developed for the treatment of symptoms caused by cancer or cancer treatment); and (v) products with a publicly available European Public Assessment Report (EPAR) of the initial marketing authorization or EPAR—refusal public assessment report (further referred to as EPAR).

EPARs were used to collect general information [drug name, active substance, mechanism of action, therapeutic indication, and cancer severity (advanced cancer, non-advanced cancer, or both in case of multiple indications)], information on genotoxicity studies [genotoxicity studies performed yes/no, type of genotoxicity studies performed, outcome of genotoxicity studies, and information on follow-up genotoxicity studies (performed yes/no, type of study, results)], and information on clinical studies (HVs used yes/no, type of clinical trial using HVs, dose of anticancer drug used in HV study, and justification for performing clinical trials in HVs). Genotoxicity studies were classified in three categories: full package, partial package, and no studies performed. The full package was defined as genotoxicity testing composing of a bacterial gene mutation study (Ames test), an in vitro cytogenic assay (test on either chromosomal damage in mammalian cells/in vitro micronucleus assay/in vitro mouse lymphoma assay), and an in vivo cytogenic assay (test for chromosomal damage in blood or bone marrow). A partial package was assigned to a drug if one or two of the studies mentioned in the full genotoxicity program were performed. No studies performed include anticancer drugs that had no available genotoxicity data at the time of the MAA. The genotoxic profile of each drug was assessed based on positive (equivocal results were considered as positive) or negative results of the bacterial mutation (Ames) assay, in vitro cytogenic assays, and in vivo cytogenic assays.

## Results

We identified 71 MAAs for small molecule anticancer drugs with a positive or negative CHMP opinion in the EU between January 2010 and December 2019. General characteristics of the anticancer drugs are summarized in Table 1. Of the 71 MAAs, 64 MAAs (90%) were for a new active substance, whereas 6 MAAs (10%) were for a known active substance. Forty-four MAAs (62%) were for the treatment of advanced cancers, 26 MAAs (37%) were for the treatment of non-advanced cancers and 1 MAA (1%) was for the treatment of both advanced and non-advanced cancers, as the MAA was approved for multiple indications. Most anticancer drugs were indicated for the treatment of blood cancer (*n* = 19; 27%), lung cancer (*n* = 9; 13%), and skin cancer (*n* = 8; 11%). Sixty-four MAAs (90%) received a positive CHMP opinion, whereas 7 MAAs (10%) received a negative CHMP opinion. Forty-eight applicants (67%) enrolled HVs in their clinical trials. Thirty three of these MAAs (69%) were for the treatment of advanced cancers, whereas 14 of these MAAs (29%) were for the treatment of non-advanced cancers, and 1 (2%) was for the treatment of both advanced and non-advancer cancers. Twenty-three applicants (33%) enrolled only patients in their clinical trials. Eleven of these MAAs (48%) were for the treatment of advanced cancers, while 12 of these MAAs (52%) were for the treatment of non-advanced cancers. Sixty-three applicants (89%) performed the full package of genotoxicity studies to support their MAA, while 7 applicants (10%) performed a partial package of genotoxicity studies to support their MAA. One applicant (1%) did not perform any genotoxicity studies as they developed a radionuclide, which in general is considered to be genotoxic and carcinogenic. Thirty two (45%) of the anticancer drugs had at least one positive finding in the standard battery of genotoxicity tests.

**Table 1:** General Characteristics of Small Molecule Anticancer Drugs with a CHMP Opinion Between January 2010 and December 2019 (*n* = 71)

Variable	Number of MAAs (%)^a^
Therapeutic indication	
Blood cancer	19 (27%)
Brain tumors	1 (1%)
Breast cancer	7 (10%)
GI tract cancer	6 (8%)
Infantile hemangioma	1 (1%)
Kidney cancer	5 (7%)
Lung cancer	9 (13%)
Neuroendocrine tumors	1 (1%)
NTRK-fusion cancer	1 (1%)
Ovarian and peritoneal cancer	4 (6%)
Prostate cancer	6 (8%)
Skin cancer	8 (11%)
Thyroid cancer	3 (4%)
Active substance	
New active substance	64 (90%)
Known active substance	7 (10%)
HVs enrolled in clinical trials	
Yes	48 (67%)
Cancer severity	
Advanced cancer	33 (69%)
Non-advanced cancer	14 (29%)
Both advanced and non-advanced cancer	1 (2%)
No	23 (33%)
Cancer severity	
Advanced cancer	11 (48%)
Non-advanced cancer	12 (52%)
Both advanced and non-advanced cancer	0
Advanced cancer	
Yes	44 (62%)
No	26 (37%)
Both	1 (1%)
CHMP opinion	
Positive	64 (90%)
Negative	7 (10%)
Genotoxicity program	
Full package	63 (89%)
Partial package	7 (10%)
No studies performed	1 (1%)
Drugs with at least one positive finding in the genotoxicity program	32 (45%)

*CHMP* Committee for Medicinal Products for Human Use, *GI* gastrointestinal, *HV* healthy volunteers, *MAA* marketing authorization application, *NTRK* neurotrophic tropomyosin receptor kinase

^a^Percentages may not total 100 due to rounding

### Genotoxicity Profile of Anticancer Drugs Administered to HVs

Forty-eight applicants enrolled HVs in their clinical trials (Table 1). Forty-seven applicants (98%) performed a full package of genotoxicity studies to determine whether their compound had a genotoxic potential (Table 2). One applicant (2%) only performed a partial package of genotoxicity tests (Table 2). A positive genotoxic finding was observed in at least one genotoxicity study for 12 anticancer drugs (26%) that were administered to HVs in clinical trials (Table 2).

**Table 2:** Characteristics of Anticancer Drugs with Phase I Clinical Trials Conducted in Healthy Volunteers (*n* = 48)

Variable	Number of MAAs (%)
Genotoxicity program	
Full package	47 (98%)
Partial package	1 (2%)
No studies performed	0
Drugs with at least one positive finding in the genotoxicity program	12 (26%)

*MAA* marketing authorization application

Of the 12 anticancer drugs with a positive finding in their genotoxicity studies, 11 anticancer drugs were kinase inhibitors, and 1 anticancer drug was a photosensitizer/vascular disruptor (Table 3). Eleven applicants performed a full package of genotoxicity tests, whereas 1 applicant only performed a partial package of genotoxicity tests (Table 3). One anticancer drug (Vanflyta) had a positive finding in the bacterial reverse mutation assay (Ames test), 8 anticancer drugs had a positive finding in the in vitro cytogenic assay, and 9 anticancer drugs had a positive finding in the in vivo cytogenic assay (Table 3). Follow-up genotoxicity tests were performed by 6 applicants, while the other applicants did not conduct any follow-up studies outside the standard battery of genotoxicity studies (Table 3). For three anticancer drugs, a genotoxic potential was not excluded based on the genotoxicity studies performed (Table 3). Vanflyta was positive in the Ames test, and an equivocal result was observed in the in vivo micronucleus test (a significant increase in micronucleated immature erythrocytes, which fell in the historical control rage). Because of the equivocal result, the CHMP advised the applicant to perform a follow-up genotoxicity test (toxicological transgenic rodent mutation assay) to provide more conclusive data on the genotoxic potential of Vanflyta. Results of this study were not provided in the EPAR. Vanflyta received a negative CHMP opinion, as the CHMP considered that the efficacy of the medicinal product was not sufficiently demonstrated. Another anticancer drug, Tookad, showed a weak potential to induce clastogenicity when illuminated by ultraviolet light. However, illumination by ultraviolet light was not performed on HVs, and therefore, the anticancer drug could be safely administered to HVs. Finally, genotoxicity studies showed that the anticancer drug Xospata had the potential to induce micronuclei in mice at doses higher than 65 mg/kg/day. No information was provided on the duration of the in vivo micronucleus test, but Xospata was only administered as a single dose of 40 mg to HVs.

**Table 3:** Genotoxicity Profile of Anticancer Drugs with a Positive Genotoxic Finding Administered to Healthy Volunteers (*n* = 12)

Product name (active substance)	Mechanism of action	Genotoxicity program	Bacterial reverse mutation assay (Ames)	In vitro cytogenic assay	In vivo cytogenic assay	Follow-up genotoxicity test	Conclusion of genotoxicity studies
Alecensa (alectinib)	Kinase inhibitor	Full package	Negative	Positive	Positive	Mechanistic in vitro and in vivo micronucleus tests combined with centromere analysis	Non-mutagenic Non-clastogenic Aneugenic at doses higher than human exposure dose
Alunbrig (brigatinib)	Kinase inhibitor	Full package	Negative	Equivocal	Positive	Mechanistic micronucleus test	Non-mutagenic Non-clastogenic. Aneugenic at doses higher than human exposure dose
Ibrance (palbociclib)	Kinase inhibitor	Full package	Negative	Positive	Positive	Kinetochore staining analysis	Non-mutagenic Non-clastogenic. Aneugenic at doses higher than human exposure dose
Inlyta (axitinib)	Kinase inhibitor	Full package	Negative	Negative	Positive	None performed	Non-mutagenic Non-clastogenic. Aneugenic at doses higher than human exposure dose
Lorviqua (lorlatinib)	Kinase inhibitor	Full package	Negative	Positive	Positive	Centromere analysis	Non-mutagenic Non-clastogenic. Aneugenic at doses higher than human exposure dose
Tookad (padeliporfin)	Photosensitizer; Vascular disrupting agent	Partial package	Negative	Weakly Positive	Not performed	None performed	Non-mutagenic Weak potential to induce clastogenicity when illuminated by ultraviolet light
Vizimpro (dacomitinib)	Kinase inhibitor	Full package	Negative	Weakly Positive	Negative	None performed	Non-mutagenic Clastogenic in vitro Non-clastogenic in vivo Not expected to be genotoxic at human exposure dose
Xalkori (crizotinib)	Kinase inhibitor	Full package	Negative	Positive	Positive	Kinetochore staining analysis	Non-mutagenic Aneugenic at doses higher than human exposure dose
Xospata (gilteritinib fumarate)	Kinase inhibitor	Full package	Negative	Negative	Positive	None performed	Non-mutagenic Potential to induce micronuclei in mice at doses higher than 65 mg/kg/day
Zydelig (idelasilib)	Kinase inhibitor	Full package	Negative	Negative	Positive	None performed	Non-mutagenic Non-clastogenic Minor chromosomal effects at the high dose in the rat study are most likely due to the pharmacological effect on PI3K kinases
Zykadia (ceratinib)	Kinase inhibitor	Full package	Negative	Positive	Negative	In vivo test analysis	Non-mutagenic Non-clastogenic
Vanflyta (quizartinib dihydrochloride)	Kinase inhibitor	Full package	Positive	Negative	Equivocal	Toxicological transgenic rodent mutation assay advised	Unclear Toxicological transgenic rodent mutation assay required to further investigate genotoxic potential

*PI3K* Phosphoinositide 3-kinase

### Anticancer Drug Dose Administered to HVs

For some compounds with a genotoxic potential, a safety margin was mentioned in the EPAR, which is based on the recommended dose in the summary of product characteristics (SmPC). In HV studies, the administered dose is often higher than the dose recommended in the SmPC. Therefore, we compared the dose of anticancer drugs with a positive genotoxic finding that was administered to HVs with the recommended dose in the SmPC to see whether HVs received a higher dose without safety margin. For 10 of the 12 anticancer drugs, the highest dose that was administered to HVs was below or equal to the recommended dose as stated in the product information (Table 4). Two anticancer drugs were administered at a higher dose to HVs than the recommended dose in the product information (Table 4). The first anticancer drug, Tookad, was administered at a single dose of 15 mg/kg to HVs, whereas the recommended dose at the time of MAA was a single dose of 3.66 mg/kg (Table 4). A genotoxic potential was, however, only observed when Tookad was illuminated by ultraviolet (Table 3). As no illumination of Tookad was performed on HVs, there was no explicit harm to HVs. The second anticancer drug, Zydelig, was administered to HVs at a total dose of 400 mg per day, versus 150 mg twice daily as recommended at the time of MAA (Table 4). Genotoxicity studies showed minor chromosomal effects at a high dose (2000 mg/kg) in a rat micronucleus study, but this effect was attributed to the mechanism of action of the active substance and a genotoxic potential could be excluded. There was, therefore, no explicit harm of administrating the higher dose to HVs.

**Table 4:** Comparison of Drug Dose Administered to HVs with the Recommended Dose as Listed in the Product Information of Anticancer Drugs with a Positive Genotoxic Finding

Product name (active substance)	Dose administered to HVs higher than recommended dose in SmPC (Yes/no)	Highest dose administered to HVs	Recommended dose SmPC
Alecensa (alectinib)	No	600 mg per day	600 mg twice daily (1200 mg daily)
Alunbrig (brigatinib)	No	180 mg per day	90 mg once daily for 7 days, then 180 mg once daily
Ibrance (palbociclib)	No	125 mg per day	125 mg once daily for 21 consecutive days followed by 7 days off treatment (Schedule 3/1)
Inlyta (axitinib)	No	5 mg per day	5 mg twice daily (10 mg daily)
Lorviqua (lorlatinib)	No	100 mg per day	100 mg once daily
Tookad (padeliporfin)	Yes	Single dose of 15 mg/kg	Single dose of 3.66 mg/kg
Vizimpro (dacomitinib)	No	45 mg per day	45 mg once daily
Xalkori (crizotinib)	No	250 mg per day	250 mg twice daily (500 mg daily)
Xospata (gilteritinib fumarate)	No	Single dose of 40 mg	120 mg once daily
Zydelig (idelasilib)	Yes	400 mg per day	150 mg twice daily (300 mg daily)
Zykadia (ceratinib)	No	750 mg per day	450 mg once daily with food; 750 mg once daily without food
Vanflyta (quizartinib dihydrochloride)	No	26.5 mg per day	26.5 mg or 53 mg per day

*HVs* healthy volunteers, *mg* milligram, *SmPC* summary of product characteristics

## Discussion

At the moment, little is known regarding how often HV studies are performed during the development of anticancer drugs. In this study, we showed that between January 2010 and December 2019, 48 applicants for anticancer drugs enrolled HVs in clinical trials. Most of the anticancer drugs administered to HVs were molecularly targeted drugs, in particular kinase inhibitors. This is in line with other studies that indicated that scientific advances in cancer research led to other types of drugs with a better safety profile, allowing the inclusion of HVs in clinical trials of anticancer drugs [6, 7]. Twelve of the anticancer drugs were administered to HVs despite a positive genotoxic finding in the standard battery of genotoxicity tests. However, harm to HVs was not expected at the doses applied to the HVs.

Almost two third of the anticancer drugs included in our study were indicated for advanced cancers. Although genotoxicity studies are not essential to support clinical trials for therapeutics intended to treat patients with advanced cancer, genotoxicity studies should be performed prior to the submission of an MAA [5]. This provides an explanation why the vast majority of applicants still conducted genotoxicity studies. In addition, we showed that HVs were more often included in clinical trials for advanced cancers than for non-advanced cancers. Genotoxicity studies should be performed prior to the inclusion of HVs in clinical trials to ensure that HVs are not exposed to immediate harm, providing another explanation why so many applicants performed genotoxicity studies. Moreover, data derived from genotoxicity studies are regarded as a substitute for long-term carcinogenicity studies in early drug development [1, 4, 5]. This becomes particularly important when HVs are included in clinical trials [6]. Almost all applicants (98%) that conducted clinical trials in HVs performed a full package of genotoxicity studies. The genotoxicity program covers three important endpoints essential in the genetic toxicology screening of investigational medicinal products: gene mutation (changes in sequence of bases), chromosome mutation (structural alteration), and genome mutation (numerical chromosome alteration) [3, 12, 13]. Negative results in the full package or partial package, as we showed for 55% of all anticancer drugs would, therefore, provide sufficient assurance of a lack of genotoxicity [12, 13].

In our study, twelve anticancer drugs were administered to HVs despite positive findings in the genotoxicity core battery studies. When we analyzed the genotoxic profile of these anticancer drugs, we found the highest number of positive genotoxic findings in the in vivo cytogenic assay, followed by the in vitro cytogenic assay. We only observed one positive finding in the Ames test. Follow-up testing is recommended when there is a positive finding in any of the tests of the genotoxic battery [3]. The appropriate follow-up test depends on which tests showed a positive genotoxic finding. Assuming that the Ames test is negative, when there is a positive finding in the in vitro cytogenic assay, typically an in vivo cytogenic assay is recommended, or an in vivo micronucleus assay when following up a potential for chromosome loss [3]. If there is an increase in micronuclei in vivo*,* all toxicological data should be evaluated to determine whether a non-genotoxic effect could be the cause or a contributing factor. In addition, mechanistic evaluation should be performed to determine whether the increase is due to chromosome loss or chromosome breakage, or whether there is a threshold exposure where chromosome loss is not expected [3]. Which genotoxicity studies should be performed to include HVs in clinical trials does not differ between anticancer drugs and other drugs. Once a genotoxic risk to HVs can be excluded, HVs may be included in clinical trials for both anticancer drugs and other drugs.

We showed that the applicants of five out of nine products (Alecensa, Alunbrig, Ibrance, Lorviqua, Xalkori) performed follow-up genotoxicity tests after a positive in vivo cytogenic assay, which excluded a genotoxic potential at the human exposure dose. Applicants of two products (Inlyta, Xospata) were able to exclude a genotoxic potential at the human exposure dose without performing follow-up genotoxicity studies. One applicant (Zydelig) could exclude a genotoxic potential, as the minor chromosomal effects observed at high doses in the rat study were attributed to the mechanism of action of the active substance. The final applicant (Vanflyta) showed a positive Ames test and a slight, but statistically significant, increase in the incidence of micronucleated immature erythrocytes in a 28-days micronucleus study in rats, although none of the data fell outside the control group. These data were considered equivocal by the CHMP, and therefore, they recommended that a toxicological transgenic rodent mutation assay should be conducted to further investigate the genotoxicity potential of the anticancer drug. No data of the follow-up study were published in the EPAR, most likely because Vanflyta received a negative CHMP opinion, as the CHMP considered that the efficacy of the medicinal product was not sufficiently demonstrated, and a follow-up study was therefore not performed.

Most of the anticancer drugs in our study that were administered to HVs despite a positive genotoxic finding were kinase inhibitors. A recent study indicated that molecularly targeted drugs, including kinase inhibitors, are often negative in the Ames test and positive in the in vitro micronucleus test. This is because kinase inhibitors tend to be specific to mammalian targets and inhibit off-target kinases, including those functional in chromosomal segregation [14]. We observed a high number of positive genotoxic fining in both in vitro and in vivo cytogenic tests.

In our study, we observed that HVs were mainly included in phase I clinical trials for the investigation of PK and PD (including food effect studies, drug-drug interaction studies, bioequivalence studies, bioavailability studies, and mass balance studies), as well as the investigation of safety and tolerability of single or multiple doses of the anticancer (data not shown). For most anticancer drugs, the dose administered to HVs was below or similar to the recommended dose stated in the SmPC. For two anticancer drugs, the dose administered to HVs was higher than the recommended dose as stated in the SmPC. For Tookad, the administration of the drug at the higher dose did not result in a risk for HVs, as a genotoxic potential was only observed after illumination by ultraviolet light, which was not applied for the HVs. For the second anticancer drug, Zydelig, a genotoxic potential was excluded by the applicant, as the positive result in the in vivo micronucleus study could be attributed to the mechanism of action of the active substance. Therefore, there was no explicit harm of administrating the higher dose to HVs.

A limitation of our study was that it was based on publicly available information in EPARs. This information may be limited, as EPARs only contain summarized data of the EMA of the full dossier from the applicant [10]. A rationale for performing a certain study was not always provided. Moreover, it was not possible to retrieve information regarding the timing of certain types of studies from the EPAR, thus, we were not able to investigate the stage of the development of the anticancer drugs when genotoxicity studies were performed.

## Conclusion

Our study showed that 48 anticancer drugs with a CHMP opinion between January 2010 and December 2019 were studied in HVs. Twelve anticancer drugs were administered to HVs despite positive findings in the genotoxic battery tests. As recommended by current regulatory guidelines, applicants performed systematic and extensive genotoxicity screening, taking into account the totality of all findings for the assessment of the genotoxic potential of the anticancer drugs. The absence of genotoxic risks to the cell system at the human exposure dose was demonstrated, and therefore, these drugs did not pose any safety concerns for HVs. In conclusion, our study suggests that the current regulatory framework for studying the genotoxic potential of anticancer drugs is sufficient to exclude immediate harm to HVs.
